# Supplementary figures and images for: Diet prevents the expansion of segmented filamentous bacteria and ileo-colonic inflammation in a model of Crohn’s disease
Source: Microbiome. 2023 Mar 31;11:66. doi: 10.1186/s40168-023-01508-y (PMC10064692; doi:10.1186/s40168-023-01508-y)

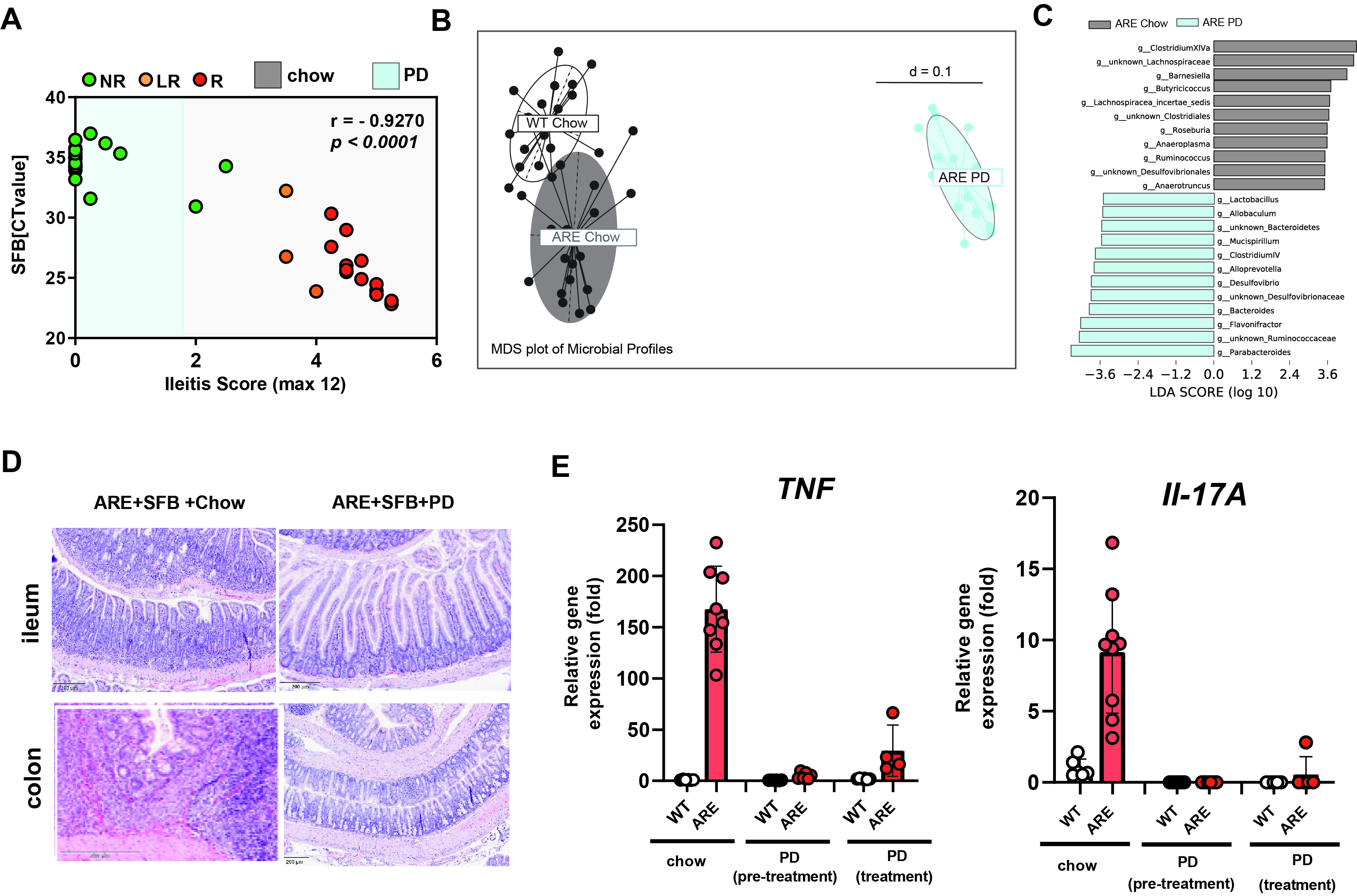

Supplement: Supplementary file 2 — Additional file 1: Supplementary Figure S1. SFB abundance correlates with ileitis severity in SPF-house Tnf ΔARE (A) SPF-housed 8-week-old ARE and WT mice were cohoused with SFB-monoassociated NOD-SCID mice for 10 weeks (B) Litter and cage effect on ileitis development in SPF-housed Tnf ΔARE mice. Squares represent males; circles indicate females. Green, orange, and red symbols indicate Tnf ΔARE mice at 18-week endpoint with no (score 0), low (score <4) and high (score >4) ileitis histopathological score, respectively; grey symbols indicate WT littermates that do not develop ileitis; white symbols indicate male mice of unknown ileitis status. Each cage is delineated with the brackets below and a cage number. (C) Quantitative Analysis of SFB in recolonized Tnf ΔARE mice (F0, F1, F2). Color-code represents the severity of inflammation as described above. CT value >30 is regarded as non-specificity threshold. (D) Ileitis scores of recolonized 18-weeks-old Tnf ΔARE mice including 2 breeding generations (F0, F1, F2). Mice are color-coded based on inflammation severity with green (score 0); orange (score <4) (orange); and red (score >4). (E) Cladogram obtained from Linear discriminant analysis effect size (LEfSe) analysis of taxonomic profiling using 16S rRNA gene sequencing of intestinal microbiota in WT and Tnf ΔARE mice. (F) Comparison of relative abundance of bacterial genera between WT and Tnf ΔARE mice using LEfSe analysis. Taxa meeting an LDA significant threshold 2 are shown, taxa enriched in Tnf ΔARE mice (red) and taxa enriched in WT mice (blue). (G) Representative H&E-stained tissue sections from distal ileum, caecum, and proximal colon of Tnf ΔARE mice colonized with single bacterial strains (Alistipes, Lactobacillus murinus and E. coli LF82) or with MIBAC, a minimal consortium of 7 bacterial strains showing no signs of inflammation. Supplementary Figure S2. Enhanced numbers of IL-17- and IFNg-expressing CD4-positive cells as well as neutrophilic granulocytes [file 40168_2023_1508_MOESM1_ESM.zip › 10.Suppl FIG 4 FIN.tif]

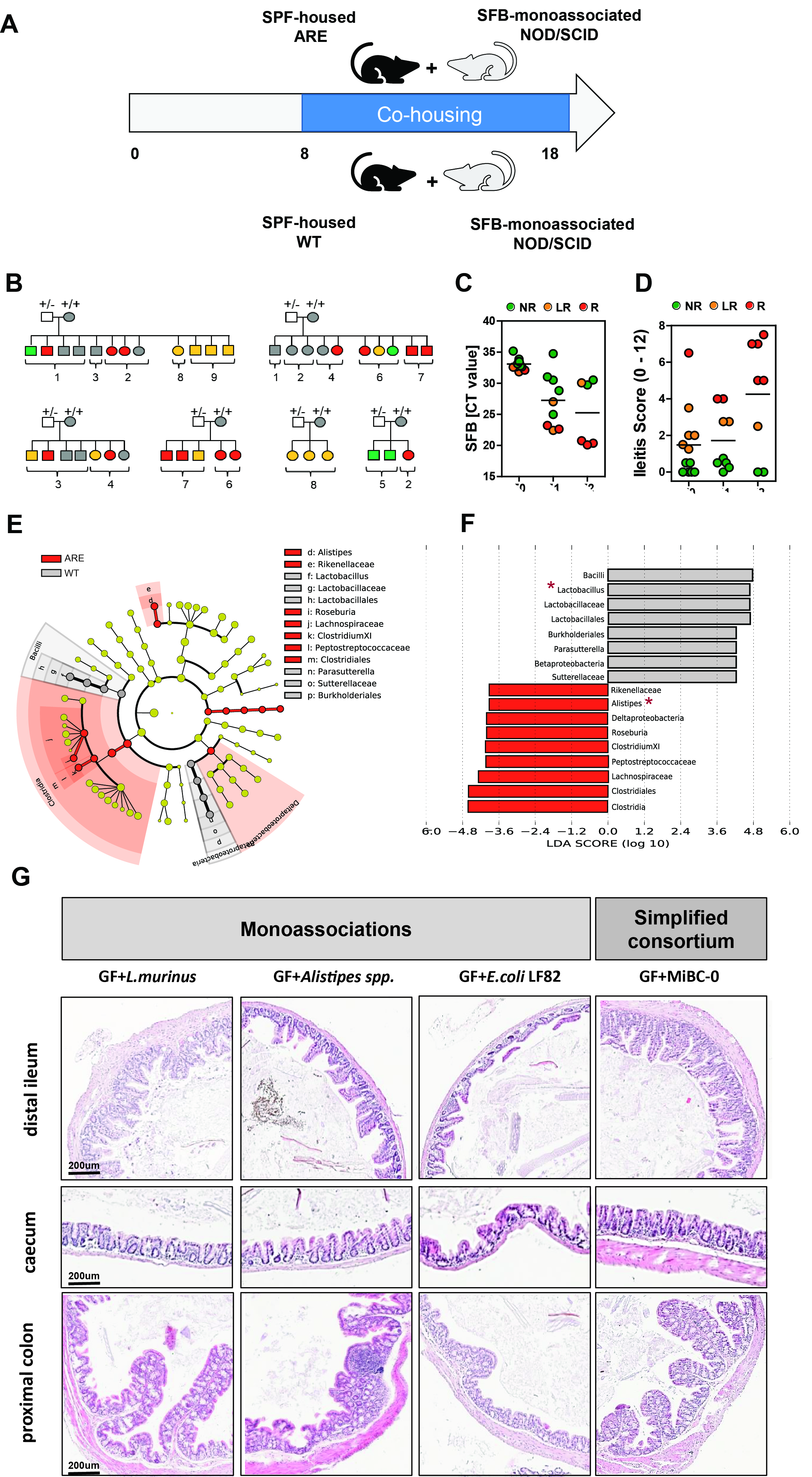

Supplement: Supplementary file 2 — Additional file 1: Supplementary Figure S1. SFB abundance correlates with ileitis severity in SPF-house Tnf ΔARE (A) SPF-housed 8-week-old ARE and WT mice were cohoused with SFB-monoassociated NOD-SCID mice for 10 weeks (B) Litter and cage effect on ileitis development in SPF-housed Tnf ΔARE mice. Squares represent males; circles indicate females. Green, orange, and red symbols indicate Tnf ΔARE mice at 18-week endpoint with no (score 0), low (score <4) and high (score >4) ileitis histopathological score, respectively; grey symbols indicate WT littermates that do not develop ileitis; white symbols indicate male mice of unknown ileitis status. Each cage is delineated with the brackets below and a cage number. (C) Quantitative Analysis of SFB in recolonized Tnf ΔARE mice (F0, F1, F2). Color-code represents the severity of inflammation as described above. CT value >30 is regarded as non-specificity threshold. (D) Ileitis scores of recolonized 18-weeks-old Tnf ΔARE mice including 2 breeding generations (F0, F1, F2). Mice are color-coded based on inflammation severity with green (score 0); orange (score <4) (orange); and red (score >4). (E) Cladogram obtained from Linear discriminant analysis effect size (LEfSe) analysis of taxonomic profiling using 16S rRNA gene sequencing of intestinal microbiota in WT and Tnf ΔARE mice. (F) Comparison of relative abundance of bacterial genera between WT and Tnf ΔARE mice using LEfSe analysis. Taxa meeting an LDA significant threshold 2 are shown, taxa enriched in Tnf ΔARE mice (red) and taxa enriched in WT mice (blue). (G) Representative H&E-stained tissue sections from distal ileum, caecum, and proximal colon of Tnf ΔARE mice colonized with single bacterial strains (Alistipes, Lactobacillus murinus and E. coli LF82) or with MIBAC, a minimal consortium of 7 bacterial strains showing no signs of inflammation. Supplementary Figure S2. Enhanced numbers of IL-17- and IFNg-expressing CD4-positive cells as well as neutrophilic granulocytes [file 40168_2023_1508_MOESM1_ESM.zip › 5.Suppl FIG 1 FIN.tif]

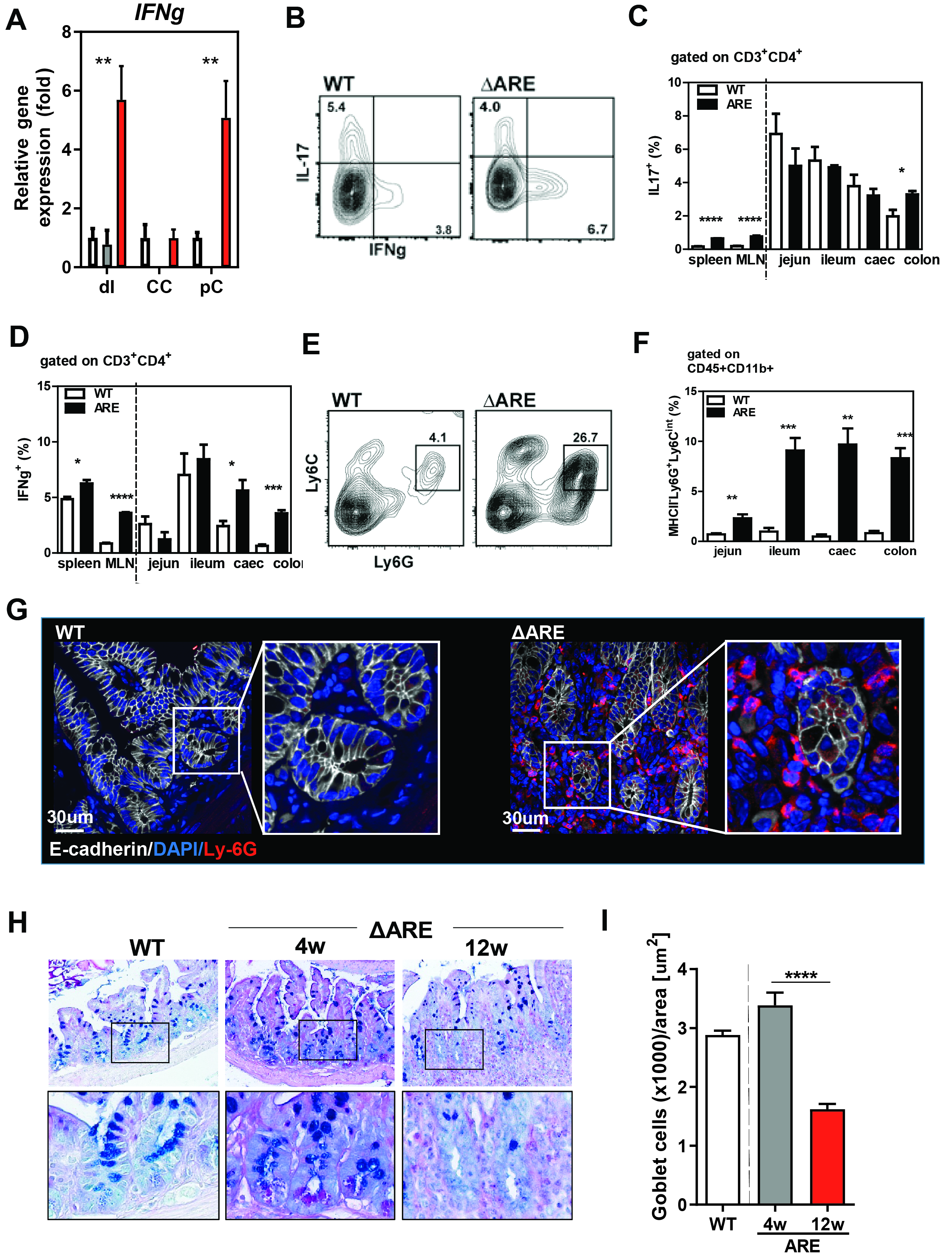

Supplement: Supplementary file 2 — Additional file 1: Supplementary Figure S1. SFB abundance correlates with ileitis severity in SPF-house Tnf ΔARE (A) SPF-housed 8-week-old ARE and WT mice were cohoused with SFB-monoassociated NOD-SCID mice for 10 weeks (B) Litter and cage effect on ileitis development in SPF-housed Tnf ΔARE mice. Squares represent males; circles indicate females. Green, orange, and red symbols indicate Tnf ΔARE mice at 18-week endpoint with no (score 0), low (score <4) and high (score >4) ileitis histopathological score, respectively; grey symbols indicate WT littermates that do not develop ileitis; white symbols indicate male mice of unknown ileitis status. Each cage is delineated with the brackets below and a cage number. (C) Quantitative Analysis of SFB in recolonized Tnf ΔARE mice (F0, F1, F2). Color-code represents the severity of inflammation as described above. CT value >30 is regarded as non-specificity threshold. (D) Ileitis scores of recolonized 18-weeks-old Tnf ΔARE mice including 2 breeding generations (F0, F1, F2). Mice are color-coded based on inflammation severity with green (score 0); orange (score <4) (orange); and red (score >4). (E) Cladogram obtained from Linear discriminant analysis effect size (LEfSe) analysis of taxonomic profiling using 16S rRNA gene sequencing of intestinal microbiota in WT and Tnf ΔARE mice. (F) Comparison of relative abundance of bacterial genera between WT and Tnf ΔARE mice using LEfSe analysis. Taxa meeting an LDA significant threshold 2 are shown, taxa enriched in Tnf ΔARE mice (red) and taxa enriched in WT mice (blue). (G) Representative H&E-stained tissue sections from distal ileum, caecum, and proximal colon of Tnf ΔARE mice colonized with single bacterial strains (Alistipes, Lactobacillus murinus and E. coli LF82) or with MIBAC, a minimal consortium of 7 bacterial strains showing no signs of inflammation. Supplementary Figure S2. Enhanced numbers of IL-17- and IFNg-expressing CD4-positive cells as well as neutrophilic granulocytes [file 40168_2023_1508_MOESM1_ESM.zip › 6.Suppl FIG 2 FIN.tif]

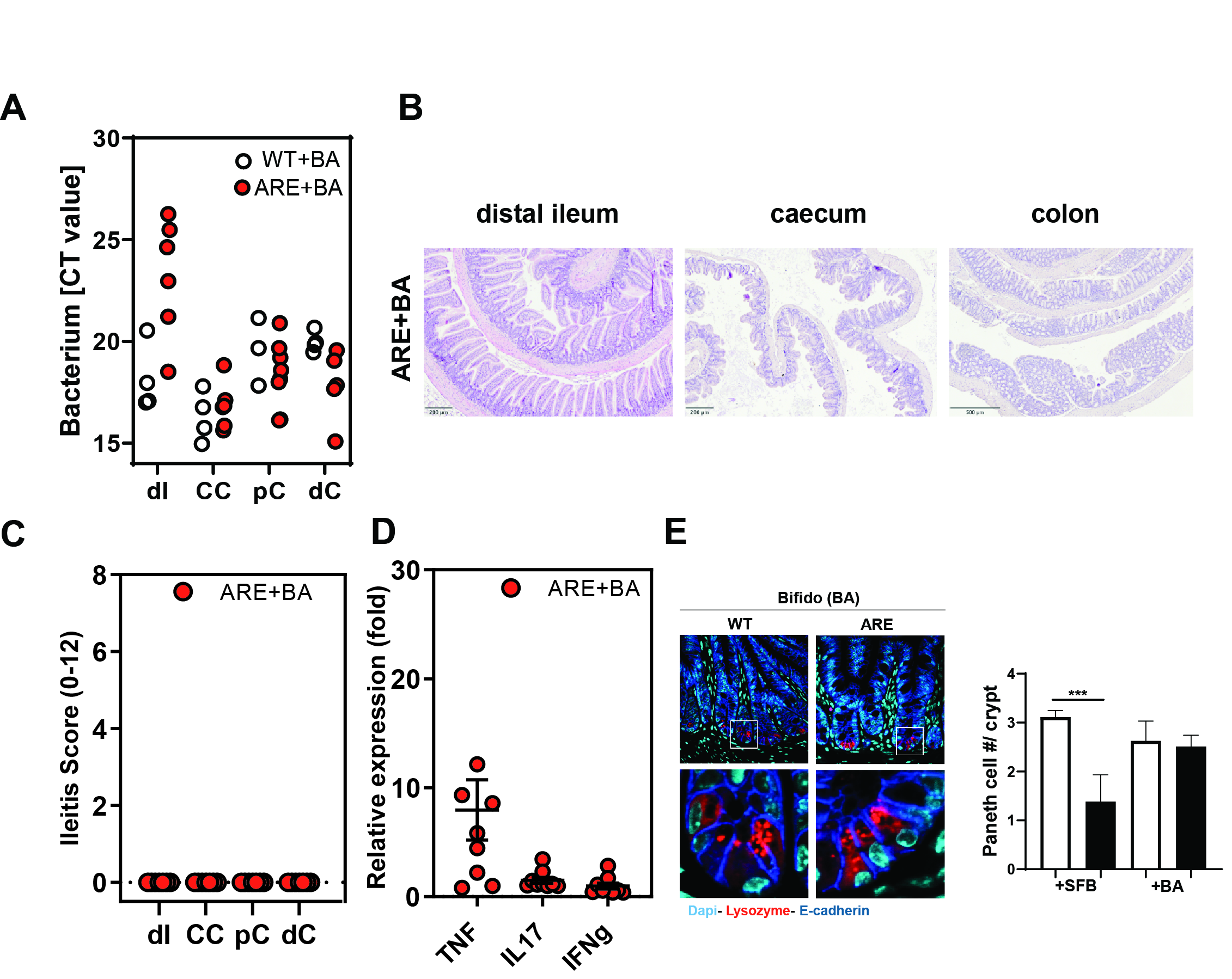

Supplement: Supplementary file 2 — Additional file 1: Supplementary Figure S1. SFB abundance correlates with ileitis severity in SPF-house Tnf ΔARE (A) SPF-housed 8-week-old ARE and WT mice were cohoused with SFB-monoassociated NOD-SCID mice for 10 weeks (B) Litter and cage effect on ileitis development in SPF-housed Tnf ΔARE mice. Squares represent males; circles indicate females. Green, orange, and red symbols indicate Tnf ΔARE mice at 18-week endpoint with no (score 0), low (score <4) and high (score >4) ileitis histopathological score, respectively; grey symbols indicate WT littermates that do not develop ileitis; white symbols indicate male mice of unknown ileitis status. Each cage is delineated with the brackets below and a cage number. (C) Quantitative Analysis of SFB in recolonized Tnf ΔARE mice (F0, F1, F2). Color-code represents the severity of inflammation as described above. CT value >30 is regarded as non-specificity threshold. (D) Ileitis scores of recolonized 18-weeks-old Tnf ΔARE mice including 2 breeding generations (F0, F1, F2). Mice are color-coded based on inflammation severity with green (score 0); orange (score <4) (orange); and red (score >4). (E) Cladogram obtained from Linear discriminant analysis effect size (LEfSe) analysis of taxonomic profiling using 16S rRNA gene sequencing of intestinal microbiota in WT and Tnf ΔARE mice. (F) Comparison of relative abundance of bacterial genera between WT and Tnf ΔARE mice using LEfSe analysis. Taxa meeting an LDA significant threshold 2 are shown, taxa enriched in Tnf ΔARE mice (red) and taxa enriched in WT mice (blue). (G) Representative H&E-stained tissue sections from distal ileum, caecum, and proximal colon of Tnf ΔARE mice colonized with single bacterial strains (Alistipes, Lactobacillus murinus and E. coli LF82) or with MIBAC, a minimal consortium of 7 bacterial strains showing no signs of inflammation. Supplementary Figure S2. Enhanced numbers of IL-17- and IFNg-expressing CD4-positive cells as well as neutrophilic granulocytes [file 40168_2023_1508_MOESM1_ESM.zip › 7.Extended Suppl FIG 2 FIN.tif]

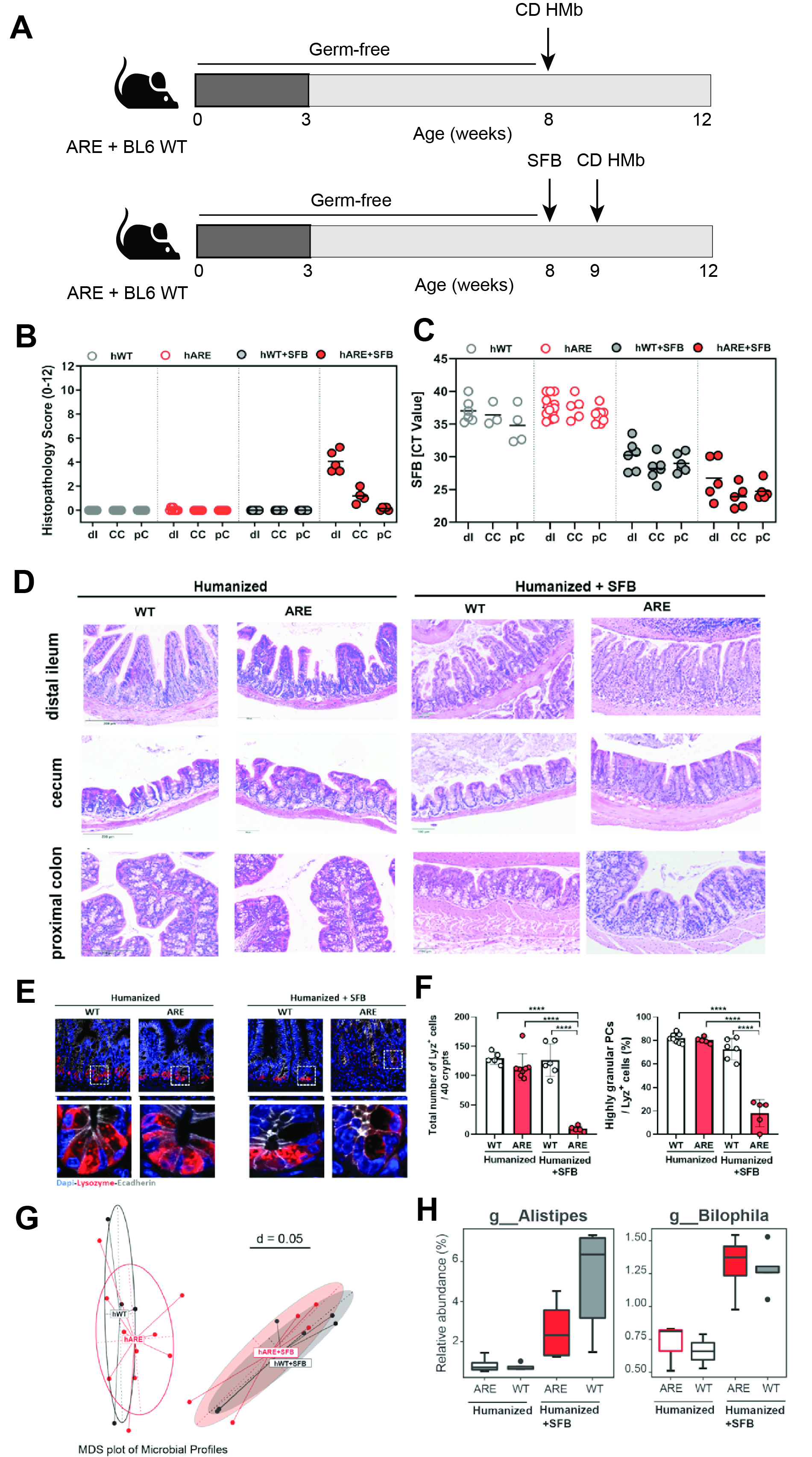

Supplement: Supplementary file 2 — Additional file 1: Supplementary Figure S1. SFB abundance correlates with ileitis severity in SPF-house Tnf ΔARE (A) SPF-housed 8-week-old ARE and WT mice were cohoused with SFB-monoassociated NOD-SCID mice for 10 weeks (B) Litter and cage effect on ileitis development in SPF-housed Tnf ΔARE mice. Squares represent males; circles indicate females. Green, orange, and red symbols indicate Tnf ΔARE mice at 18-week endpoint with no (score 0), low (score <4) and high (score >4) ileitis histopathological score, respectively; grey symbols indicate WT littermates that do not develop ileitis; white symbols indicate male mice of unknown ileitis status. Each cage is delineated with the brackets below and a cage number. (C) Quantitative Analysis of SFB in recolonized Tnf ΔARE mice (F0, F1, F2). Color-code represents the severity of inflammation as described above. CT value >30 is regarded as non-specificity threshold. (D) Ileitis scores of recolonized 18-weeks-old Tnf ΔARE mice including 2 breeding generations (F0, F1, F2). Mice are color-coded based on inflammation severity with green (score 0); orange (score <4) (orange); and red (score >4). (E) Cladogram obtained from Linear discriminant analysis effect size (LEfSe) analysis of taxonomic profiling using 16S rRNA gene sequencing of intestinal microbiota in WT and Tnf ΔARE mice. (F) Comparison of relative abundance of bacterial genera between WT and Tnf ΔARE mice using LEfSe analysis. Taxa meeting an LDA significant threshold 2 are shown, taxa enriched in Tnf ΔARE mice (red) and taxa enriched in WT mice (blue). (G) Representative H&E-stained tissue sections from distal ileum, caecum, and proximal colon of Tnf ΔARE mice colonized with single bacterial strains (Alistipes, Lactobacillus murinus and E. coli LF82) or with MIBAC, a minimal consortium of 7 bacterial strains showing no signs of inflammation. Supplementary Figure S2. Enhanced numbers of IL-17- and IFNg-expressing CD4-positive cells as well as neutrophilic granulocytes [file 40168_2023_1508_MOESM1_ESM.zip › 8.Suppl FIG 3 FIN.tif]

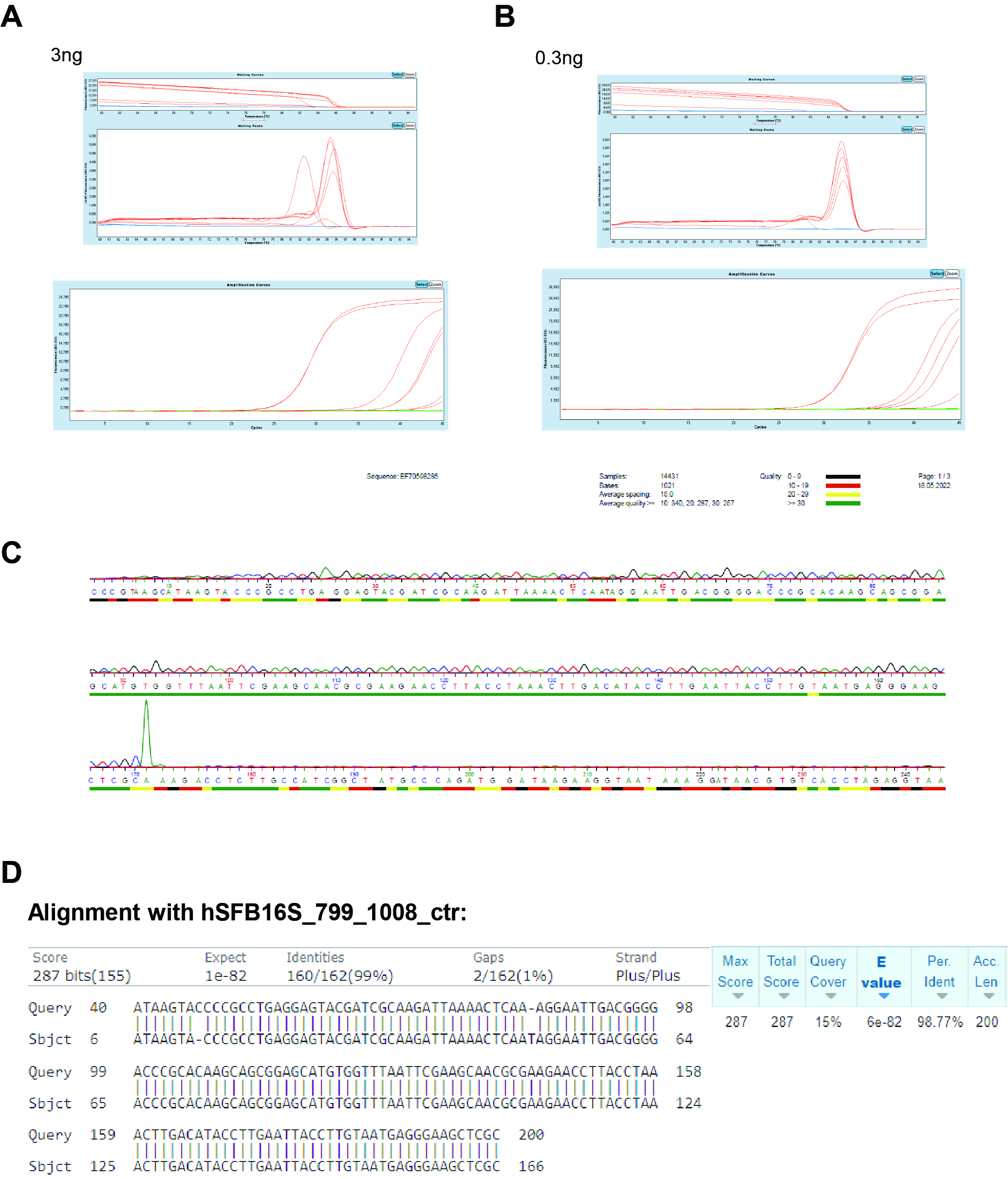

Supplement: Supplementary file 2 — Additional file 1: Supplementary Figure S1. SFB abundance correlates with ileitis severity in SPF-house Tnf ΔARE (A) SPF-housed 8-week-old ARE and WT mice were cohoused with SFB-monoassociated NOD-SCID mice for 10 weeks (B) Litter and cage effect on ileitis development in SPF-housed Tnf ΔARE mice. Squares represent males; circles indicate females. Green, orange, and red symbols indicate Tnf ΔARE mice at 18-week endpoint with no (score 0), low (score <4) and high (score >4) ileitis histopathological score, respectively; grey symbols indicate WT littermates that do not develop ileitis; white symbols indicate male mice of unknown ileitis status. Each cage is delineated with the brackets below and a cage number. (C) Quantitative Analysis of SFB in recolonized Tnf ΔARE mice (F0, F1, F2). Color-code represents the severity of inflammation as described above. CT value >30 is regarded as non-specificity threshold. (D) Ileitis scores of recolonized 18-weeks-old Tnf ΔARE mice including 2 breeding generations (F0, F1, F2). Mice are color-coded based on inflammation severity with green (score 0); orange (score <4) (orange); and red (score >4). (E) Cladogram obtained from Linear discriminant analysis effect size (LEfSe) analysis of taxonomic profiling using 16S rRNA gene sequencing of intestinal microbiota in WT and Tnf ΔARE mice. (F) Comparison of relative abundance of bacterial genera between WT and Tnf ΔARE mice using LEfSe analysis. Taxa meeting an LDA significant threshold 2 are shown, taxa enriched in Tnf ΔARE mice (red) and taxa enriched in WT mice (blue). (G) Representative H&E-stained tissue sections from distal ileum, caecum, and proximal colon of Tnf ΔARE mice colonized with single bacterial strains (Alistipes, Lactobacillus murinus and E. coli LF82) or with MIBAC, a minimal consortium of 7 bacterial strains showing no signs of inflammation. Supplementary Figure S2. Enhanced numbers of IL-17- and IFNg-expressing CD4-positive cells as well as neutrophilic granulocytes [file 40168_2023_1508_MOESM1_ESM.zip › 9.Extended Suppl FIG 3 FIN.tif]
