# Supplementary material for: Diet prevents the expansion of segmented filamentous bacteria and ileo-colonic inflammation in a model of Crohn’s disease
Source: Microbiome. 2023 Mar 31;11:66. doi: 10.1186/s40168-023-01508-y (PMC10064692; doi:10.1186/s40168-023-01508-y)
Supplement: Supplementary file 3 — Additional file 2: Supplementary Table S1. Composition of chow, purified and fiber-rich diet. Supplementary Table S2. Description and metadata of patient mucosal biopsy samples screened for SFB presence. Supplementary Table S3. Minimal bacterial consortium (MIBAC) composition. [file 40168_2023_1508_MOESM2_ESM.docx]

|  | **Chow Diet** | **PD** | **FRD** |
| --- | --- | --- | --- |
| **Carbohydrates [E%]** | **61** | **64** | **61-64** |
| **Fat [E%]** | **12** | **13** | **12-13** |
|  | **soybean oil** | **soybean oil** | **soybean oil** |
| **Protein [E%]** | **27** | **23** | **23-27** |
| **Fiber [g/kg]** | **Not specified; prob. 200-300** | **50** | **200-300** |
|  | **Cellulose, hemicellulose, lignin (not specified)** | **Cellulose** | **Mixed Complex (simulating fiber content in Chow Diet)** |
| **Simple**  **Sugar [g/kg]** | **Not specified and not added** | **60** | **60** |

**Supplementary Table S1:** Composition of chow, purified and fiber-rich diet

**Supplementary Table S2:** Description and metadata of patient mucosal biopsy samples screened for SFB presence.

| **#SampleID** | **Cohort** | **Disease** | **Location** |
| --- | --- | --- | --- |
| H7 | Biotherapy | CD | Colon |
| H6 | Biotherapy | CD | Colon |
| I1 | Biotherapy | CD | Ileum |
| I2 | Biotherapy | CD | Ileum |
| H9 | Biotherapy | CD | Ileum |
| B2 | Biotherapy | CD | Ileum |
| A3 | Biotherapy | Poc | Ileum |
| A5 | Biotherapy | CD | Ileum |
| B4 | Biotherapy | CD | Ileum |
| B6 | Biotherapy | CD | Ileum |
| B2 | Biotherapy | Poc | Ileum |
| C6 | Biotherapy | Poc | Ileum |
| A1 | Biotherapy | Poc | Ileum |
| A6 | Biotherapy | CD | Ileum |
| D9 | Biotherapy | CD | Ileum |
| E1 | Biotherapy | CD | Ileum |
| C5 | Biotherapy | Poc | Ileum |
| B5 | Biotherapy | CD | Ileum |
| G5 | Biotherapy | Poc | Ileum |
| B1 | Biotherapy | Poc | Ileum |
| G7 | Biotherapy | Poc | Ileum |
| D8 | Biotherapy | CD | Ileum |
| B3 | Biotherapy | CD | Ileum |
| A7 | Biotherapy | CD | Ileum |
| C9 | Biotherapy | CD | Ileum |
| G6 | Biotherapy | Poc | Ileum |
| A7 | Biotherapy | CD | Ileum |
| D5 | Biotherapy | Poc | Ileum |
| C4 | Biotherapy | Poc | Ileum |
| E5 | Biotherapy | CD | Ileum |
| D8 | Biotherapy | CD | Ileum |
| B5 | Biotherapy | CD | Ileum |
| A2 | Biotherapy | Poc | Ileum |
| E4 | Biotherapy | CD | Ileum |
| E3 | Biotherapy | CD | Ileum |
| D3 | Biotherapy | Poc | Ileum |
| E2 | Biotherapy | CD | Ileum |
| D4 | Biotherapy | Poc | Ileum |
| B4 | Biotherapy | CD | Ileum |
| B6 | Biotherapy | CD | Ileum |
| B1 | Biotherapy | CD | Ileum |
| B8 | Biotherapy | CD | Ileum |
| D5 | Biotherapy | Poc | Ileum |
| B7 | Biotherapy | CD | Ileum |
| E6 | Biotherapy | CD | Ileum |
| A6 | Biotherapy | CD | Rectum |
| A8 | Biotherapy | CD | Rectum |
| E5 | Biotherapy | CD | Rectum |
| A9 | Biotherapy | CD | Rectum |
| E6 | Biotherapy | CD | Rectum |
| E7 | Biotherapy | UC | Rectum |
| D7 | Biotherapy | CD | Rectum |
| E7 | Biotherapy | UC | Rectum |
| B9 | Biotherapy | UC | Rectum |
| D4 | Biotherapy | UC | Rectum |
| C8 | Biotherapy | CD | Rectum |
| D6 | Biotherapy | CD | Rectum |
| B8 | Biotherapy | UC | Rectum |
| C7 | Biotherapy | UC | Rectum |
| C1 | Biotherapy | UC | Rectum |
| C2 | Biotherapy | UC | Rectum |
| A5 | Biotherapy | CD | Rectum |
| D3 | Biotherapy | UC | Rectum |
| E8 | Biotherapy | UC | Rectum |
| B2 | Biotherapy | CD | Rectum |
| G3 | Biotherapy | UC | Rectum |
| D5 | Biotherapy | UC | Rectum |
| A9 | Biotherapy | UC | Rectum |
| F8 | Biotherapy | UC | Rectum |
| G2 | Biotherapy | UC | Rectum |
| C7 | Biotherapy | CD | Rectum |
| C3 | Biotherapy | UC | Rectum |
| F9 | Biotherapy | UC | Rectum |
| C9 | Biotherapy | CD | Rectum |
| B3 | Biotherapy | CD | Rectum |
| G4 | Biotherapy | UC | Rectum |
| G1 | Biotherapy | UC | Rectum |
| G8 | Biotherapy | CD | Rectum |
| G7 | Biotherapy | CD | Rectum |
| D8 | Biotherapy | CD | Right Colon |
| D7 | Biotherapy | CD | Right Colon |
| D7 | Biotherapy | CD | Right Colon |
| C8 | Biotherapy | CD | Right Colon |
| D6 | Biotherapy | CD | Right colon or Rectum |
| I3 | Biotherapy | UC | Sigmoid |
| C8 | Biotherapy | UC | Sigmoid |
| B7 | Biotherapy | UC | Sigmoid |
| C5 | Biotherapy | CD | Sigmoid |
| B7 | Biotherapy | CD | Sigmoid |
| A3 | Biotherapy | CD | Sigmoid |
| I7 | Biotherapy | CD | Sigmoid |
| A6 | Biotherapy | UC | Sigmoid |
| B8 | Biotherapy | CD | Sigmoid |
| C6 | Biotherapy | UC | Sigmoid |
| A7 | Biotherapy | UC | Sigmoid |
| C9 | Biotherapy | UC | Sigmoid |
| H2 | Biotherapy | CD | Sigmoid |
| D4 | Biotherapy | UC | Sigmoid |
| F4 | Biotherapy | UC | Sigmoid |
| B3 | Biotherapy | CD | Sigmoid |
| F5 | Biotherapy | UC | Sigmoid |
| D2 | Biotherapy | UC | Sigmoid |
| I5 | Biotherapy | CD | Sigmoid |
| H3 | Biotherapy | CD | Sigmoid |
| H9 | Biotherapy | UC | Sigmoid |
| E1 | Biotherapy | UC | Sigmoid |
| H8 | Biotherapy | CD | Sigmoid |
| H4 | Biotherapy | CD | Sigmoid |
| F6 | Biotherapy | UC | Sigmoid |
| I3 | Biotherapy | CD | Sigmoid |
| F3 | Biotherapy | UC | Sigmoid |
| D1 | Biotherapy | UC | Sigmoid |
| E9 | Biotherapy | UC | Sigmoid |
| E2 | Biotherapy | UC | Sigmoid |
| B6 | Biotherapy | UC | Sigmoid |
| I1 | Biotherapy | UC | Sigmoid |
| D3 | Biotherapy | UC | Sigmoid |
| A5 | Biotherapy | UC | Sigmoid |
| F8 | Biotherapy | UC | Sigmoid |
| H6 | Biotherapy | UC | Sigmoid |
| G4 | Biotherapy | UC | Sigmoid |
| G3 | Biotherapy | UC | Sigmoid |
| C4 | Biotherapy | CD | Sigmoid |
| F1 | Biotherapy | UC | Sigmoid |
| F9 | Biotherapy | UC | Sigmoid |
| A9 | Biotherapy | UC | Sigmoid |
| E3 | Biotherapy | UC | Sigmoid |
| H5 | Biotherapy | UC | Sigmoid |
| G9 | Biotherapy | UC | Sigmoid |
| B5 | Biotherapy | CD | Sigmoid |
| H8 | Biotherapy | UC | Sigmoid |
| A8 | Biotherapy | UC | Sigmoid |
| I4 | Biotherapy | CD | Sigmoid |
| H4 | Biotherapy | UC | Sigmoid |
| F2 | Biotherapy | UC | Sigmoid |
| F5 | Biotherapy | UC | Sigmoid |
| D1 | Biotherapy | CD | Sigmoid |
| E9 | Biotherapy | CD | Sigmoid |
| E8 | Biotherapy | CD | Sigmoid |
| C6 | Biotherapy | CD | Sigmoid |
| B4 | Biotherapy | CD | Sigmoid |
| C2 | Biotherapy | UC | Sigmoid |
| B9 | Biotherapy | CD | Sigmoid |
| A8 | Biotherapy | UC | Sigmoid |
| D2 | Biotherapy | UC | Sigmoid |
| B1 | Biotherapy | UC | Sigmoid |
| A1 | Biotherapy | UC | Sigmoid |
| G1 | Biotherapy | UC | Sigmoid |
| H1 | Biotherapy | UC | Sigmoid |
| I2 | Biotherapy | UC | Sigmoid |
| F2 | Biotherapy | UC | Sigmoid |
| F6 | Biotherapy | UC | Sigmoid |
| C3 | Biotherapy | CD | Sigmoid |
| H2 | Biotherapy | UC | Sigmoid |
| H7 | Biotherapy | UC | Sigmoid |
| C1 | Biotherapy | UC | Sigmoid |
| C4 | Biotherapy | CD | Sigmoid |
| F7 | Biotherapy | UC | Sigmoid |
| C1 | Biotherapy | CD | Sigmoid |
| I7 | Biotherapy | CD | Sigmoid |
| G9 | Biotherapy | UC | Sigmoid |
| D1 | Biotherapy | CD | Sigmoid |
| I8 | Biotherapy | CD | Sigmoid |
| C7 | Biotherapy | UC | Sigmoid |
| G2 | Biotherapy | UC | Sigmoid |
| I6 | Biotherapy | CD | Sigmoid |
| D2 | Biotherapy | CD | Sigmoid |
| H3 | Biotherapy | UC | Sigmoid |
| I8 | Biotherapy | CD | Sigmoid |
| I5 | Biotherapy | CD | Sigmoid |
| F3 | Biotherapy | UC | Sigmoid |
| C3 | Biotherapy | UC | Sigmoid |
| A2 | Biotherapy | UC | Sigmoid |
| F1 | Biotherapy | CD | Sigmoid |
| A4 | Biotherapy | CD | Sigmoid |
| G5 | Biotherapy | CD | Sigmoid |
| F7 | Biotherapy | UC | Sigmoid |
| G8 | Biotherapy | UC | Sigmoid |
| I9 | Biotherapy | UC | Sigmoid |
| A4 | Biotherapy | UC | Sigmoid |
| G6 | Biotherapy | CD | Sigmoid |
| A3 | Biotherapy | UC | Sigmoid |
| C5 | Biotherapy | CD | Sigmoid |
| I9 | Biotherapy | CD | Transverse Colon |
| A2 | Biotherapy | CD | Transverse Colon |
| A1 | Biotherapy | CD | Transverse Colon |
| B107 | Control | Control | ASCENDING COLON |
| B113 | Control | Control | ASCENDING COLON |
| B115 | Control | Control | ASCENDING COLON |
| B119 | Control | Control | ASCENDING COLON |
| B120 | Control | Control | ASCENDING COLON |
| B123 | Control | Control | ASCENDING COLON |
| B126 | Control | Control | ASCENDING COLON |
| B129 | Control | Control | ASCENDING COLON |
| B132 | Control | Control | ASCENDING COLON |
| B137 | Control | Control | ASCENDING COLON |
| B140 | Control | Control | ASCENDING COLON |
| B154 | Control | Control | ASCENDING COLON |
| B157 | Control | Control | ASCENDING COLON |
| B160 | Control | Control | ASCENDING COLON |
| B168 | Control | Control | ASCENDING COLON |
| B171 | Control | Control | ASCENDING COLON |
| B177 | Control | Control | ASCENDING COLON |
| B178 | Control | Control | ASCENDING COLON |
| B181 | Control | Control | ASCENDING COLON |
| B122 | Control | Control | ILEUM |
| B125 | Control | Control | ILEUM |
| B128 | Control | Control | ILEUM |
| B131 | Control | Control | ILEUM |
| B134 | Control | Control | ILEUM |
| B135 | Control | Control | ILEUM |
| B138 | Control | Control | ILEUM |
| B152 | Control | Control | ILEUM |
| B155 | Control | Control | ILEUM |
| B158 | Control | Control | ILEUM |
| B175 | Control | Control | ILEUM |
| B180 | Control | Control | ILEUM |
| B183 | Control | Control | ILEUM |
| B110 | Control | Control | SIGMOID COLON |
| B114 | Control | Control | SIGMOID COLON |
| B116 | Control | Control | SIGMOID COLON |
| B121 | Control | Control | SIGMOID COLON |
| B124 | Control | Control | SIGMOID COLON |
| B127 | Control | Control | SIGMOID COLON |
| B130 | Control | Control | SIGMOID COLON |
| B133 | Control | Control | SIGMOID COLON |
| B136 | Control | Control | SIGMOID COLON |
| B139 | Control | Control | SIGMOID COLON |
| B153 | Control | Control | SIGMOID COLON |
| B156 | Control | Control | SIGMOID COLON |
| B159 | Control | Control | SIGMOID COLON |
| B167 | Control | Control | SIGMOID COLON |
| B170 | Control | Control | SIGMOID COLON |
| B176 | Control | Control | SIGMOID COLON |
| B179 | Control | Control | SIGMOID COLON |
| B182 | Control | Control | SIGMOID COLON |
| B006 | HSCT | CD | ASCENDING COLON |
| B008 | HSCT | CD | ASCENDING COLON |
| B011 | HSCT | CD | ASCENDING COLON |
| B014 | HSCT | CD | ASCENDING COLON |
| B017 | HSCT | CD | ASCENDING COLON |
| B018 | HSCT | CD | ASCENDING COLON |
| B021 | HSCT | CD | ASCENDING COLON |
| B023 | HSCT | CD | ASCENDING COLON |
| B025 | HSCT | CD | ASCENDING COLON |
| B028 | HSCT | CD | ASCENDING COLON |
| B030 | HSCT | CD | ASCENDING COLON |
| B033 | HSCT | CD | ASCENDING COLON |
| B036 | HSCT | CD | ASCENDING COLON |
| B040 | HSCT | CD | ASCENDING COLON |
| B042 | HSCT | CD | ASCENDING COLON |
| B053 | HSCT | CD | ASCENDING COLON |
| B061 | HSCT | CD | ASCENDING COLON |
| B063 | HSCT | CD | ASCENDING COLON |
| B087 | HSCT | CD | ASCENDING COLON |
| B089 | HSCT | CD | ASCENDING COLON |
| B103 | HSCT | CD | ASCENDING COLON |
| B105 | HSCT | CD | ASCENDING COLON |
| B143 | HSCT | CD | ASCENDING COLON |
| B148 | HSCT | CD | ASCENDING COLON |
| B066 | HSCT | CD | DESCENDING COLON |
| B069 | HSCT | CD | DESCENDING COLON |
| B070 | HSCT | CD | DESCENDING COLON |
| B073 | HSCT | CD | DESCENDING COLON |
| B074 | HSCT | CD | DESCENDING COLON |
| B075 | HSCT | CD | DESCENDING COLON |
| B076 | HSCT | CD | DESCENDING COLON |
| B078 | HSCT | CD | DESCENDING COLON |
| B081 | HSCT | CD | DESCENDING COLON |
| B084 | HSCT | CD | DESCENDING COLON |
| B085 | HSCT | CD | DESCENDING COLON |
| B092 | HSCT | CD | DESCENDING COLON |
| B095 | HSCT | CD | DESCENDING COLON |
| B097 | HSCT | CD | DESCENDING COLON |
| B099 | HSCT | CD | DESCENDING COLON |
| B100 | HSCT | CD | DESCENDING COLON |
| B151 | HSCT | CD | DESCENDING COLON |
| B163 | HSCT | CD | DESCENDING COLON |
| B187 | HSCT | CD | DESCENDING COLON |
| B005 | HSCT | CD | ILEUM |
| B007 | HSCT | CD | ILEUM |
| B012 | HSCT | CD | ILEUM |
| B013 | HSCT | CD | ILEUM |
| B016 | HSCT | CD | ILEUM |
| B019 | HSCT | CD | ILEUM |
| B020 | HSCT | CD | ILEUM |
| B022 | HSCT | CD | ILEUM |
| B024 | HSCT | CD | ILEUM |
| B027 | HSCT | CD | ILEUM |
| B031 | HSCT | CD | ILEUM |
| B035 | HSCT | CD | ILEUM |
| B038 | HSCT | CD | ILEUM |
| B043 | HSCT | CD | ILEUM |
| B044 | HSCT | CD | ILEUM |
| B047 | HSCT | CD | ILEUM |
| B050 | HSCT | CD | ILEUM |
| B051 | HSCT | CD | ILEUM |
| B052 | HSCT | CD | ILEUM |
| B055 | HSCT | CD | ILEUM |
| B058 | HSCT | CD | ILEUM |
| B059 | HSCT | CD | ILEUM |
| B067 | HSCT | CD | ILEUM |
| B068 | HSCT | CD | ILEUM |
| B071 | HSCT | CD | ILEUM |
| B072 | HSCT | CD | ILEUM |
| B080 | HSCT | CD | ILEUM |
| B083 | HSCT | CD | ILEUM |
| B091 | HSCT | CD | ILEUM |
| B093 | HSCT | CD | ILEUM |
| B094 | HSCT | CD | ILEUM |
| B096 | HSCT | CD | ILEUM |
| B098 | HSCT | CD | ILEUM |
| B101 | HSCT | CD | ILEUM |
| B147 | HSCT | CD | ILEUM |
| B161 | HSCT | CD | ILEUM |
| B165 | HSCT | CD | ILEUM |
| B189 | HSCT | CD | ILEUM |
| B009 | HSCT | CD | RECTUM |
| B010 | HSCT | CD | RECTUM |
| B015 | HSCT | CD | RECTUM |
| B026 | HSCT | CD | RECTUM |
| B029 | HSCT | CD | RECTUM |
| B032 | HSCT | CD | RECTUM |
| B034 | HSCT | CD | RECTUM |
| B037 | HSCT | CD | RECTUM |
| B039 | HSCT | CD | RECTUM |
| B041 | HSCT | CD | RECTUM |
| B046 | HSCT | CD | RECTUM |
| B054 | HSCT | CD | RECTUM |
| B056 | HSCT | CD | RECTUM |
| B057 | HSCT | CD | RECTUM |
| B060 | HSCT | CD | RECTUM |
| B062 | HSCT | CD | SIGMOID COLON |
| B064 | HSCT | CD | SIGMOID COLON |
| B088 | HSCT | CD | SIGMOID COLON |
| B090 | HSCT | CD | SIGMOID COLON |
| B102 | HSCT | CD | SIGMOID COLON |
| B104 | HSCT | CD | SIGMOID COLON |
| B141 | HSCT | CD | SIGMOID COLON |
| B142 | HSCT | CD | SIGMOID COLON |
| B144 | HSCT | CD | SIGMOID COLON |
| B146 | HSCT | CD | SIGMOID COLON |
| B162 | HSCT | CD | SIGMOID COLON |
| B164 | HSCT | CD | SIGMOID COLON |
| B186 | HSCT | CD | SIGMOID COLON |
| B188 | HSCT | CD | SIGMOID COLON |
| B045 | HSCT | CD | TRANSVERSE COLON |
| B048 | HSCT | CD | TRANSVERSE COLON |
| B077 | HSCT | CD | TRANSVERSE COLON |
| B079 | HSCT | CD | TRANSVERSE COLON |
| B082 | HSCT | CD | TRANSVERSE COLON |
| B086 | HSCT | CD | TRANSVERSE COLON |
| B149 | HSCT | CD | ILEUM |
| B172 | HSCT | CD | ILEUM |
| B173 | HSCT | CD | ILEUM |
| B174 | HSCT | CD | ILEUM |
| B184 | HSCT | CD | ILEUM |
| B185 | HSCT | CD | ILEUM |
| P01 | Pediatric | CD | Sigma |
| P01 | Pediatric | CD | Ileum |
| P01 | Pediatric | CD | Sigma |
| P02 | Pediatric | CD | Ileum |
| P02 | Pediatric | CD | Sigma |
| P02 | Pediatric | CD | Ileum |
| P02 | Pediatric | CD | Sigma |
| P03 | Pediatric | CD | Ileum |
| P03 | Pediatric | CD | Sigma |
| P03 | Pediatric | CD | Ileum |
| P04 | Pediatric | UC | Ileum |
| P04 | Pediatric | UC | Sigma |
| P04 | Pediatric | UC | Ileum |
| P04 | Pediatric | UC | Sigma |
| P05 | Pediatric | CD | Ileum |
| P05 | Pediatric | CD | Sigma |
| P05 | Pediatric | CD | Ileum |
| P05 | Pediatric | CD | Sigma |
| P06 | Pediatric | CD | Ileum |
| P06 | Pediatric | CD | Sigma |
| P06 | Pediatric | CD | Ileum |
| P06 | Pediatric | CD | Sigma |
| P07 | Pediatric | CD | Ileum |
| P07 | Pediatric | CD | Sigma |
| P07 | Pediatric | CD | Ileum |
| P07 | Pediatric | CD | Sigma |
| P08 | Pediatric | UC | Ileum |
| P08 | Pediatric | UC | Sigma |
| P08 | Pediatric | UC | Sigma |
| P08 | Pediatric | UC | Sigma |
| P09 | Pediatric | CD | Ileum |
| P09 | Pediatric | CD | Sigma |
| P09 | Pediatric | CD | Ileum |
| P09 | Pediatric | CD | Sigma |
| P10 | Pediatric | CD | Sigma |
| P10 | Pediatric | CD | Ileum |
| P10 | Pediatric | CD | Sigma |
| P11 | Pediatric | UC | Ileum |
| P11 | Pediatric | UC | Sigma |
| P11 | Pediatric | UC | Ileum |
| P11 | Pediatric | UC | Sigma |
| P12 | Pediatric | IBDU | Sigmoid |
| P13 | Pediatric | UC | Ileum |
| P13 | Pediatric | UC | Sigmoid |
| P14 | Pediatric | UC | Sigmoid |
| P15 | Pediatric | UC | Ileum |
| P15 | Pediatric | UC | Sigmoid |
| P16 | Pediatric | UC | Ileum |
| P16 | Pediatric | UC | Sigmoid |
| P17 | Pediatric | CD | Ileum |
| P17 | Pediatric | CD | Sigmoid |
| P18 | Pediatric | UC | Ileum |
| P18 | Pediatric | UC | Sigmoid |
| P19 | Pediatric | IBDU | Ileum |
| P19 | Pediatric | IBDU | Sigmoid |
| P20 | Pediatric | UC | Ileum |
| P21 | Pediatric | UC | Ileum |
| P21 | Pediatric | UC | Sigmoid |
| P22 | Pediatric | UC | Ileum |
| P07 | Pediatric |  | Ileum |
| P07 | Pediatric |  | Sigma |
| P07 | Pediatric |  | Ileum |
| P07 | Pediatric |  | Sigma |
| P07 | Pediatric |  | Ileum |
| P184 | Pediatric |  | Sigma |
| P184 | Pediatric |  | Ileum |
| P184 | Pediatric |  | Sigma |
| P150 | Pediatric |  | Ileum |
| P150 | Pediatric |  | Sigma |
| P150 | Pediatric |  | Cecum |
| P150 | Pediatric |  | Sigma |
| P10 | Pediatric |  | Ileum |
| P10 | Pediatric |  | Sigma |
| P10 | Pediatric |  | Sigma |
| P10 | Pediatric |  | Sigma |
| P10 | Pediatric |  | Ileum |
| P10 | Pediatric |  | Sigma |
| P10 | Pediatric |  | Sigma |
| P30 | Pediatric |  | Ileum |
| P30 | Pediatric |  | Sigma |
| P30 | Pediatric |  | Ileum |
| P30 | Pediatric |  | Sigma |
| P46 | Pediatric |  | Ileum |
| P46 | Pediatric |  | Sigma |
| P47 | Pediatric |  | Ileum |
| P47 | Pediatric |  | Sigma |
| P47 | Pediatric |  | Ileum |
| P47 | Pediatric |  | Sigma |
| P49 | Pediatric |  | Ileum |
| P49 | Pediatric |  | Sigma |
| P49 | Pediatric |  | Ileum |
| P49 | Pediatric |  | Sigma |
| P60 | Pediatric |  | Ileum |
| P60 | Pediatric |  | Ileum |
| P60 | Pediatric |  | Sigma |
| P66 | Pediatric |  | Ileum |
| P66 | Pediatric |  | Sigma |
| P66 | Pediatric |  | Ileum |
| P66 | Pediatric |  | Sigma |
| P163 | Pediatric |  | Ileum |
| P163 | Pediatric |  | Sigma |
| P163 | Pediatric |  | Ileum |
| P163 | Pediatric |  | Sigma |
| P175 | Pediatric |  | Ileum |
| P175 | Pediatric |  | Sigma |
| P175 | Pediatric |  | Ileum |
| P175 | Pediatric |  | Sigma |
| P176 | Pediatric |  | Ileum |
| P176 | Pediatric |  | Sigma |
| P176 | Pediatric |  | Ileum |
| P176 | Pediatric |  | Sigma |
| P182 | Pediatric |  | Ileum |
| P182 | Pediatric |  | Sigma |
| P182 | Pediatric |  | Ileum |
| P182 | Pediatric |  | Sigma |
| P174 | Pediatric |  | Ileum |
| P174 | Pediatric |  | Sigma |
| P174 | Pediatric |  | Ileum |
| P174 | Pediatric |  | Sigma |
| P210 | Pediatric |  | Ileum |
| P210 | Pediatric |  | Sigma |
| P210 | Pediatric |  | Sigma |
| P210 | Pediatric |  | Sigma |
| P094 | Pediatric |  | Ileum |
| P094 | Pediatric |  | Sigma |
| P094 | Pediatric |  | Ileum |
| P094 | Pediatric |  | Sigma |
| P162 | Pediatric |  | Ileum |
| P162 | Pediatric |  | Sigma |
| P162 | Pediatric |  | Ileum |
| P162 | Pediatric |  | Sigma |
| P194 | Pediatric |  | Ileum |
| P194 | Pediatric |  | Sigma |
| P194 | Pediatric |  | Ileum |
| P194 | Pediatric |  | Sigma |
| P200 | Pediatric |  | Ileum |
| P200 | Pediatric |  | Sigma |
| P201 | Pediatric |  | Ileum |
| P201 | Pediatric |  | Sigma |
| P185 | Pediatric |  | Ileum |
| P185 | Pediatric |  | Sigma |
| P185 | Pediatric |  | Sigma |
| P189 | Pediatric |  | Ileum |
| P189 | Pediatric |  | Sigma |
| P189 | Pediatric |  | Ileum |
| P189 | Pediatric |  | Sigma |
| P206 | Pediatric |  | Ileum |
| P206 | Pediatric |  | Sigma |
| P215 | Pediatric |  | Ileum |
| P215 | Pediatric |  | Sigma |
| P220 | Pediatric |  | Ileum |
| P220 | Pediatric |  | Sigma |

**Supplementary Table S3:** Minimal bacterial consortium (MIBAC) composition

| *Species* | **Original strain designation** | **DSM no.** |
| --- | --- | --- |
| ***Clostridium ramosum*** | **SRB509'-5-F-B** | **29357** |
| ***Paraclostridium bifermentans*** | **G7K1R3-PYG-90** | **29423** |
| ***Enterococcus hirae*** | **SB** | **28619** |
| ***Enterorhabdus mucosicola*** | **Mt1B8** | **19490** |
| ***Escherichia coli*** | **Mt1B1** | **28618** |
| ***Lactobacillus murinus*** | **M-6244-3B** | **28683** |
| ***Parabacteroides goldsteinii*** | **BS-C3-2** | **29187** |
